# Gender differences in clinical features and complications of infective endocarditis: 11-year experience of a single institute in Egypt

**DOI:** 10.1186/s43044-020-0039-6

**Published:** 2020-01-21

**Authors:** Ahmed Adel Elamragy, Marwa Sayed Meshaal, Amani Ali El-Kholy, Hussein Hassan Rizk

**Affiliations:** 1 Department of Cardiology, Kasr Al Aini Hospital, Faculty of Medicine, Cairo University, Cairo, 11562 Egypt; 2 Department of Clinical Pathology and Microbiology, Kasr Al Aini Hospital, Faculty of Medicine, Cairo University, Cairo, 11562 Egypt

**Keywords:** Endocarditis, Registries, Gender, Egypt

## Abstract

**Background:**

No data exists about the gender differences among patients with infective endocarditis (IE) in Egypt. The objective was to study possible gender differences in clinical profiles and outcomes of patients in the IE registry of a tertiary care center over 11 years.

**Results:**

The IE registry included 398 patients with a median age of 30 years (interquartile range, 15 years); 61.1% were males. Males were significantly older than females. Malignancy and recent culprit procedures were more common in females while chronic liver disease and intravenous drug abuse (IVDU) were more in males. IE on top of structurally normal hearts was significantly more in males (25.6% vs 13.6%, *p* = 0.005) while rheumatic valvular disease was more common in females (46.3% vs 29.9%, *p* = 0.001). There was no difference in the duration of illness before presentation to our institution. The overall complication rate was high but significantly higher in females. However, there were no significant differences in the major complications: mortality, fulminant sepsis, renal failure requiring dialysis, heart failure class III–IV, or major cerebrovascular emboli.

**Conclusion:**

In this registry, IE occurred predominantly in males. Females were significantly younger at presentation. History of recent culprit procedures was more common in females while IVDU was more common in males who had a higher incidence of IE on structurally normal hearts. The overall complication rate was higher in women. IE management and its outcomes were similar in both genders.

## Background

Gender differences and their impact on the clinical profile and outcome in cardiovascular diseases are a debatable issue in the literature [1–3]. These differences may be attributed to a variety of factors including variable comorbidities, treatment biases, or inherent physiologic differences [3]. Previous reports addressing this issue in patients with infective endocarditis (IE) did not examine its relationship with outcomes and treatment decisions systematically [2, 3]. IE occurs in males more frequently than females, with 2:1 to 9:1 ratio [4–6]. Animal models suggested a potential protective role for estrogen against endothelial damage [7, 8], whereas human studies showed that females less likely develop sepsis after traumatic hemorrhagic shock [9–11], but the exact mechanism is not fully understood. Moreover, females tend to encounter heart disease at an older age than males. Thus, younger females are relatively protected from IE predispositions. On the other hand, females have a higher incidence of comorbid conditions (e.g., diabetes mellitus (DM) and renal failure) which may complicate IE management and result in worse outcomes [12].

In Egypt, no data exists about the gender differences among patients with IE regarding clinical characteristics or prognosis. Gender biases in offering diagnostic and treatment services are alleged in rural and underprivileged areas.

## Methods

### Patient cohort

An IE specialized unit and registry were established in February 2005 in a tertiary care facility in Egypt to define the clinical profile of IE patients and improve the awareness and management of this disease entity. The registry details were previously published [13], including definitions of terms used (prosthetic valve endocarditis (PVE), intravenous drug use (IVDU)-associated IE and healthcare-associated endocarditis (HAE)), the microbiologic data (blood cultures, serologic tests, surgical specimen cultures, and histopathologic examination), and follow-up during the hospital course. The current study involves all patients with possible/definite IE [14] between February 2005 and September 2016. Transthoracic echocardiography (TTE) was performed within 24–48 h of hospital admission followed by trans-esophageal echocardiography (TEE) within another 72 h if there was a clinical indication [13]. All patients received the appropriate management protocols as recommended by the current guidelines [15–21]. Patient education on IE prophylaxis, dental care, and symptoms and signs of IE recurrence was provided upon discharge. In addition to regular patient care, the dedicated IE team organized regular meetings and seminars, distributed brochures about proper IE diagnosis and management, and arranged infection control workshops on proper blood and tissue sampling techniques. Besides, regular internal and external audit meetings were held to monitor the progress of the IE registry [13].

### Statistical analysis

Data analysis was performed by SPSS 20.0 program. Categorical data were expressed as percentages. Continuous variables were skewed and presented as median and interquartile range (IQR). Differences in categorical variables were tested by the chi-squared test or Fisher’s exact (when appropriate). The comparison of continuous variables was done using the Mann-Whitney test. Gender-specific significant univariate variables for in-hospital mortality (with *p* < 1.0) entered a stepwise conditional multivariate regression analysis to detect the most significant independent predictors with the corresponding odds ratio (OR) and 95% confidence interval (CI). Statistical significance was considered at *p* < 0.05.

## Results

### Clinical characteristics

The patient cohort included 398 patients; median age, 30 years (IQR, 24–39 years), 61.1% were males. The main clinical features and comorbidities are demonstrated in Table 1. Males were significantly older than females. Malignancy and recent culprit procedures were more common in females while chronic liver disease and IVDU were more in males. There was a trend for higher HAE in females. However, the various culprit procedures did not differ between both genders (intravenous lines, early PVE, dialysis, non-cardiac surgeries, dental procedures, or urinary catheter insertions). IE on top of structurally normal hearts was significantly more prevalent in males while rheumatic heart disease (RHD) was more common in females. The duration of illness before presentation to our institution was very long and did not differ between both genders.

**Table 1:** Clinical characteristics based on gender

	Males (*n* = 243), *N* (%)	Females (*n* = 155), *N* (%)	*p*
Age (years)	31 (24–40)	28 (23–37)	*0.002*
Comorbid conditions			
DM	13 (5.3)	7 (4.5)	0.71
Renal insufficiency	28 (11.5)	19 (12.3)	0.83
Liver disease	14 (5.5)	0 (0)	*0.001*
Malignancy	0 (0)	7 (4.5)	*0.001*
Clinical history			
Fever	198 (81.5)	137 (88.4)	0.07
Duration of febrile illness	28 (14–60)	28 (12–56)	0.76
Previous use of antibiotics	146 (60.1)	85 (54.8)	0.30
Prior IE	12 (4.9)	3 (1.9)	0.13
Drug abuse	40 (16.5)	2 (1.3)	*< 0.001*
HAE	105 (44.9)	80 (54.4)	0.069
Procedures within the last 3 months	49 (20.9)	46 (31.3)	*0.023*
Hospitalization within the last 3 months	85 (36.3)	66 (44.9)	0.096
Underlying cardiac disease	147 (60.5)	108 (69.7)	0.06
Rheumatic heart disease	70 (29.9)	68 (46.3)	*0.001*
Congenital heart disease	24 (10.3)	8 (5.4)	0.099
Degenerative valve disease	7 (3.0)	3 (2)	0.57
Normal heart	60 (25.6)	20 (13.6)	*0.005*
Hypertrophic cardiomyopathy	3 (1.3)	0 (0)	0.29
PVE	69 (28.4)	47 (30.3)	0.68

*DM* diabetes mellitus, *IE* infective endocarditis, *HAE* healthcare-associated IE, *PVE* Prosthetic valve endocarditis

### Echocardiographic findings

The main echocardiographic features are represented in Table 2. Left-sided vegetation (mitral and aortic valves) and development of ring abscesses were more common in females, whereas right-sided vegetation (namely the tricuspid valve) was more common in males.

**Table 2:** Echocardiographic features of both genders

	Males (*n* = 243), *N* (%)	Females (*n* = 155), *N* (%)	*p*
TTE diagnostic	194 (79.8)	120 (77.4)	0.57
TEE diagnostic	121 (90.3%)	72 (91.1%)	0.84
Presence of vegetation	184 (75.7)	114 (73.5)	0.63
Left-sided vegetation	138 (56.8)	102 (65.8)	0.07
Aortic valve vegetation TTE	81 (33.3)	33 (21.3)	*0.01*
Mitral valve vegetation TTE	80 (32.9)	79 (51)	*< 0.001*
Aortic valve vegetation TEE	57 (48.7)	26 (40)	0.26
Mitral valve vegetation TEE	66 (55.9)	51 (65.4)	0.19
Aortic valve vegetation TTE or TEE	93 (65.5)	42 (53.2)	0.07
Mitral valve vegetation TTE or TEE	101 (69.2)	99 (81.8)	*0.018*
Right sided vegetation	53 (21.8)	15 (9.7)	*0.002*
Pulmonary valve vegetation	3 (1.2)	3 (1.9)	0.58
Tricuspid valve vegetation	48 (19.8)	14 (9)	*0.004*
Ring abscess	32 (22.4)	20 (12.9)	*0.03*
Pericardial effusion	49 (20.2)	36 (23.2 )	0.47

*TTE* transthoracic echocardiography, *TEE* trans-esophageal echocardiography

### Microbiologic data

The rate of negative blood cultures was equally high in males and females (69.5% vs 68.4%, *p* = 0.8), and there were no gender differences in the rate of organism detection using different methods (surgical specimen culture and histology). The most common detected organisms were staphylococcal species (28.8% vs 21.3%, *p* = 0.1). There were no gender differences in all other detected organisms (streptococci, enterococci, gram-negative bacilli, fungi, or zoonotic organisms). Empirical antibiotics were also equally given in both genders (59.3% vs 55.5%, *p* = 0.5).

### Complications

**Fig. 1:**
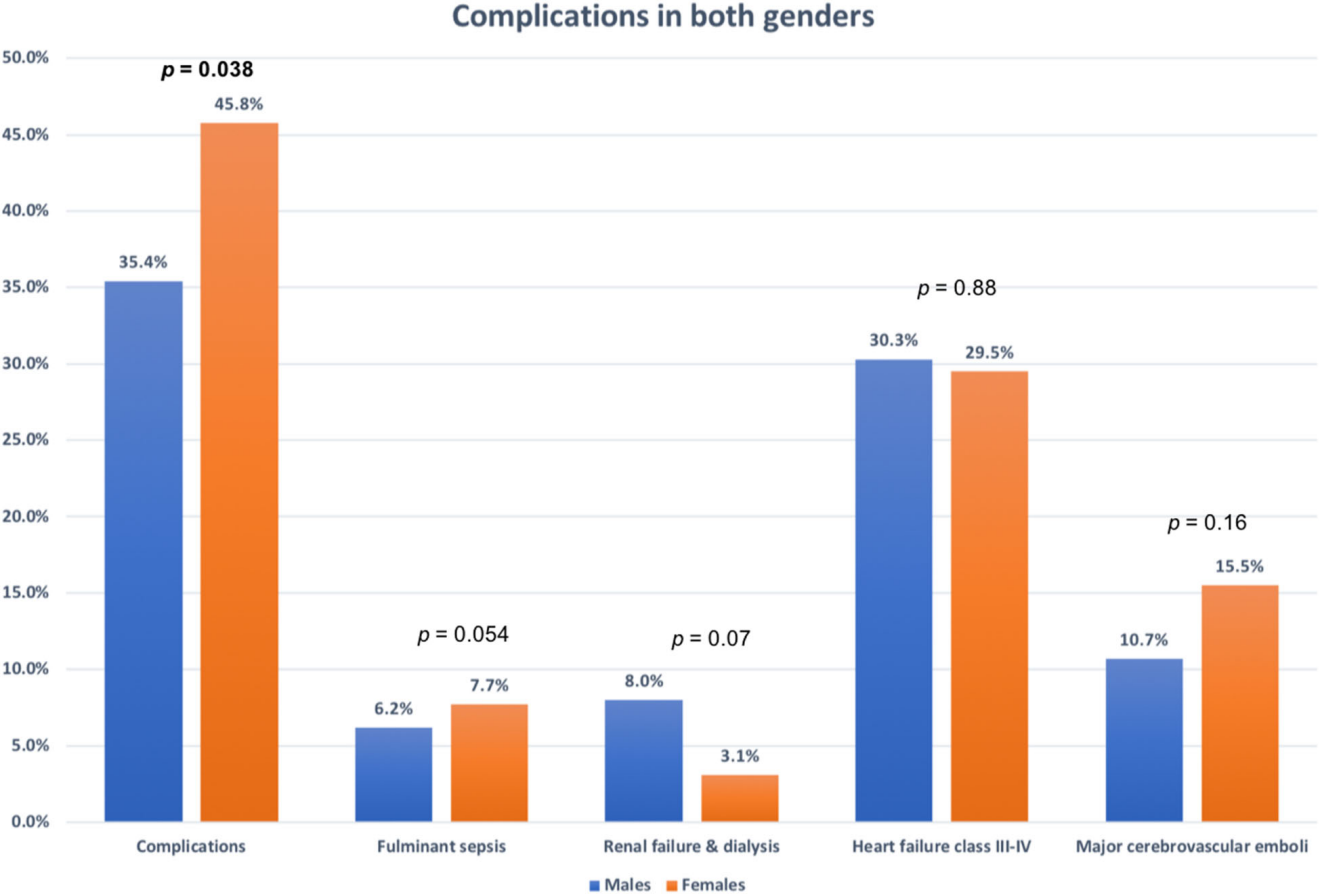
Complication rates in both genders

**Fig. 2:**
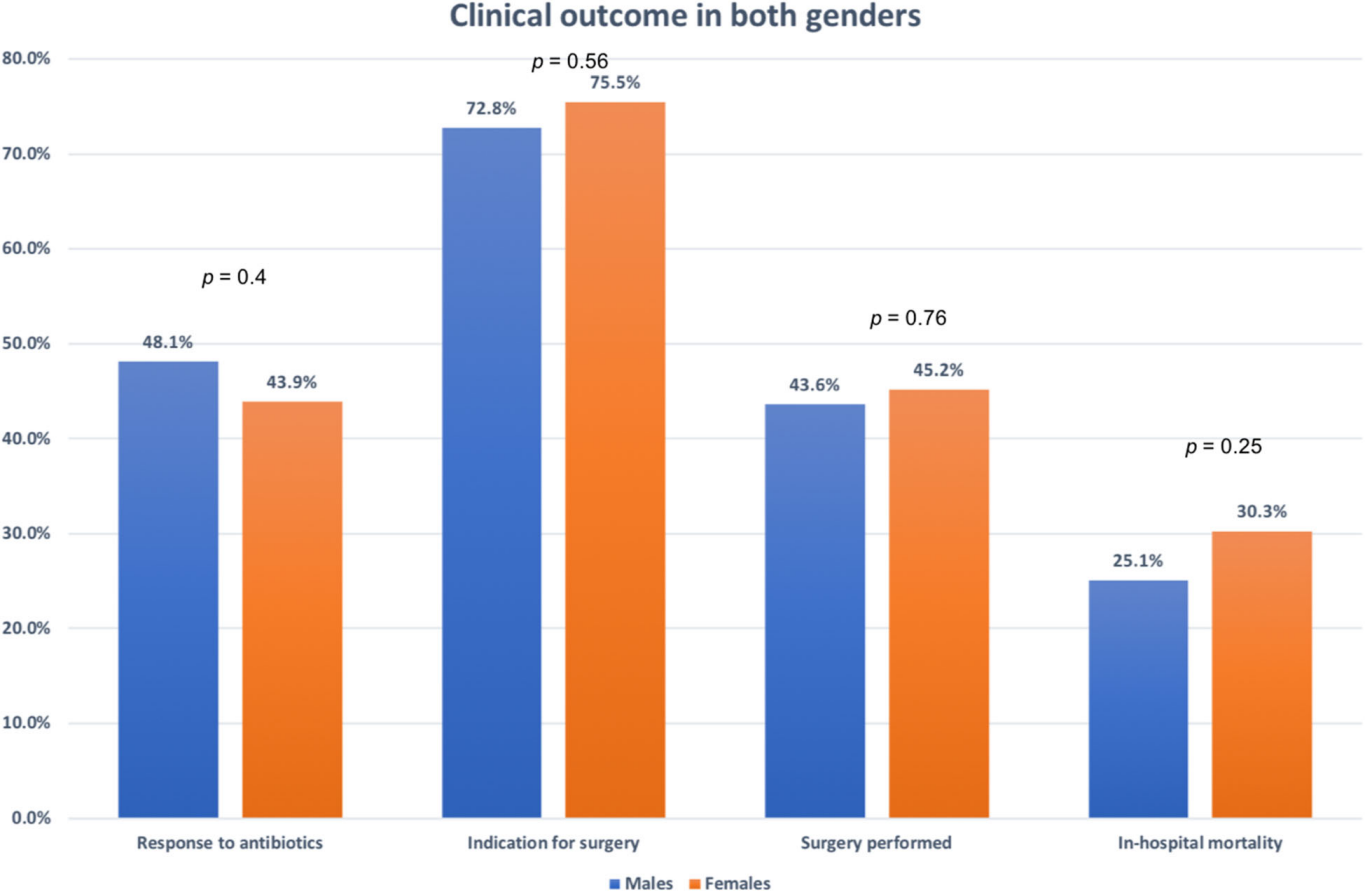
Clinical outcome in both genders

The overall complication rate was high, but it was significantly higher in females. However, the major complications (fulminant sepsis, renal failure requiring dialysis, advanced heart failure, major cerebrovascular stroke, and in-hospital mortality) were similar between both genders. Surgery was equally performed when clinically indicated (Figs. 1 and 2)

### Predictors of in-hospital mortality in both genders

Univariate predictors of in-hospital mortality are presented in Table 3. Using multivariate analysis, the most important predictors of in-hospital mortality in males were poor response to antibiotics (OR, 57.0; CI, 12.7–269.1, *p* < 0.001), fulminant sepsis (OR, 10.8; 95% CI, 1.2–98.2; *p* = 0.034), and not performing surgery when indicated (OR, 8.9; 95% CI, 3.5–23.0; *p* < 0.001). In females, poor response to antibiotics was the sole predictor of in-hospital mortality (OR, 15.1; 95% CI, 1.5–161.0; *p* = 0.022).

**Table 3:** Predictors of in-hospital mortality in both genders

	Males			Females		
Univariate analysis	Survival, *n* = 182, *N* (%)	Mortality, *n* = 61, *N* (%)	*p*	Survival, *n* = 08, *N* (%)	Mortality, *n* = 47, *N* (%)	*p*
HF NYHA III–IV	27 (14.8)	26 (42.6)	*< 0.001*	19 (17.6)	19 (40.4)	*0.002*
Fulminant sepsis	1 (0.5)	14 (23)	*< 0.001*	1 (0.9)	11 (23.4)	*< 0.001*
ARF requiring dialysis	4 (3.4)	10 (17.5)	*0.002*	0 (0)	4 (8.7)	*0.015*
Poor response to Abs	68 (37.4)	58 (95.1)	*< 0.001*	42 (38.9)	45 (95.7)	*< 0.001*
Indication for surgery	125 (68.7)	52 (85.2)	*0.012*	74 (68.5)	43 (91.5)	*0.002*
Indicated surgery not performed	33 (26.4)	38 (73.1)	*< 0.001*	22 (29.7)	25 (58.1)	*0.003*
Major cerebrovascular emboli	14 (7.7)	12 (19.7)	*0.009*	14 (13)	10 (21.3)	0.188
Embolization	42 (23.1)	27 (44.3)	*0.001*	42 (38.9)	22 (46.8)	0.36
HAE	70 (38.5)	35 (57.4)	*0.01*	51 (47.2)	29 (61.7)	*0.097*
RHD	45 (24.7)	25 (41)	*0.015*	48 (44.4)	20 (42.6)	0.83
ICH	8 (4.4)	8 (13.1)	*0.017*	4 (3.7)	6 (12.8)	*0.035*
Aortic root abscess	16 (8.8)	5 (8.2)	0.89	4 (3.7)	5 (10.6)	*0.09*
Mycotic aneurysms	10 (5.5)	3 (4.9)	1.0	5 (4.6)	6 (12.8)	*0.09*
Pericardial effusion	32 (17.6)	17 (27.9)	*0.083*	24 (22.2)	12 (25.5)	0.65
Indicated surgery not performed	8.94	3.47–23.04	*< 0.001*			

*NYHA* New York Heart Association, *ARF* acute renal failure, *Abs* antibiotics, *HAE* healthcare-associated endocarditis, *RHD* rheumatic heart disease, *ICH* intracranial hemorrhage, *OR* odds ratio, *CI* confidence interval

## Discussion

This is a subgroup analysis of an IE registry in a tertiary care facility for 11 years in which we studied the main differences in IE features and management between both genders. The main registry report and subgroup reports were previously published [13, 22–28]. Differences in offering diagnostic and therapeutic healthcare services based on gender have been a major worldwide concern over the years [29–32]. In this analysis, there were no gender differences in the management and outcome of IE patients. The response to antibiotic treatment, the need for surgical treatment, the rate of surgical interventions, and overall in-hospital mortality were similar in both genders. Previous IE studies showed worse outcomes among females [1, 33, 34], because of less surgical interventions in the active phase of the disease [34] or comorbid conditions that put females at a higher risk [1, 3].

Comorbid conditions like DM and renal insufficiency were not different between both genders. However, males suffered more from liver diseases, while females had a higher incidence of malignancy. This contrasts with a previous study [3] which showed a higher prevalence of DM, renal insufficiency, and immunosuppression among females that was translated into a worse outcome. Another study [1] also showed that renal insufficiency and immunosuppression were more common in females.

We previously reported a high rate of overall complications (39.4%) [13]. A striking feature was the significantly higher rate among females that can be attributed to the trend of higher rates of HAE which translates to highly resistant strains. Fortunately, the major life-threatening complications (advanced heart failure, fulminant sepsis, renal failure, major cerebrovascular emboli, and in-hospital mortality) did not differ between both genders. This is in contrast to a previous study [3] that showed a twofold higher rate of in-hospital mortality among females, explained by the presence of more comorbid conditions. The same study and another one [34] also showed a higher rate of surgical interventions among males. In our registry, the rate of performing surgery when indicated, the use of antibiotics, and the overall outcome were similar between both genders. This confirms the absence of gender-bias in offering medical services in our facility.

In our registry, IE occurred more commonly in males (61.1%) with a ratio of 1.6:1. This gender predilection for IE was previously reported in Saudi Arabia [35], United Kingdom (UK) [36], Japan [37], and other reports [4–6]. There are theories about a potential protective role for estrogen against endothelial damage [7, 8] and a less likelihood of developing sepsis in females [9–11], but the exact mechanism is not fully understood.

In this analysis, a history of recent culprit procedures was more common in females, who also had a trend to encounter HAE and a higher incidence of RHD. They are more exposed to health care procedures (concerned with birth control, pregnancy, and labor), which puts them at a higher risk for developing HAE, at a young age, especially when predisposed with high rates of RHD. This may explain the younger age of females with IE in this registry. On the other hand, males had a higher incidence of IE on structurally normal hearts, likely because of a higher incidence of IVDU among them, which can affect normal hearts.

This analysis revealed a very high rate of culture-negative IE (69.1%) that was equal in both genders. This may be explained by the indiscriminate use of antibiotics for the treatment of any febrile illness before obtaining blood cultures (58%). This is a common practice in our country, and it applies to both genders. This rate was also high in African countries like Algeria (56.4%) [38] and South Africa (55.3%) [39] compared to European countries like the UK (12.2%) [40] and France (9%) [41].

Right-sided valves (namely the tricuspid valve) were more commonly involved among males due to a higher incidence of IVDU. On the other hand, mitral valve involvement (detected by TTE) was more common in females, while aortic valve involvement and the development of aortic ring abscess were more common in males. This finding was previously shown in a study by Hamda et al. [42] and may be explained by the higher rate of mitral disease in females and aortic disease in males [43].

This analysis identified several issues that can be addressed to improve IE management. Medical personnel education about infection control measures and timely management of the various febrile illnesses can be translated into lower rates of HAE, culture-negative IE, and complications leading to better IE outcomes. Increasing the awareness of the health hazards of IVDU may also decrease the IE infection rates among males.

## Conclusion

In this registry, IE occurred predominantly in males. Females were significantly younger at presentation. History of recent culprit procedures was more common in females while IVDU was more common in males who had a higher incidence of IE on structurally normal hearts. The overall complication rate was higher in women. IE management and its outcomes were similar in both genders.

## Data Availability

The datasets used and/or analyzed during the current study are available from the corresponding author on reasonable request.

## Abbreviations

CI: Confidence interval
DM: Diabetes mellitus
HAE: Healthcare-associated endocarditis
IE: Infective endocarditis
IQR: Interquartile range
IVDU: Intravenous drug use
OR: Odds ratio
PVE: Prosthetic valve endocarditis
RHD: Rheumatic heart disease
TEE: Trans-esophageal echocardiography
TTE: Transthoracic echocardiography
UK: United Kingdom
